# A case of Pathological Laughter in a patient with recurrent stroke

**DOI:** 10.1192/j.eurpsy.2023.1632

**Published:** 2023-07-19

**Authors:** S. Lakhotia, M. N. Parikh

**Affiliations:** Psychiatry, BJ Medical College and Civil Hospital Ahmedabad, Ahmedabad, India

## Abstract

**Introduction:**

Stroke survivors frequently deal with neuropsychiatric sequelae - depression, anxiety and apathy being the most common ones. Pathological laughing and crying (PLC) is a post stroke condition characterized by brief, intense uncontrollable crying and/or laughing due to a neurological disorder. Prevalence of PLC post stroke has been reported to be 15-20%. Pathological laughter (PL) is commonly associated with bilateral or diffuse cerebral lesions. Ischemic injury involving the internal capsule and basal ganglia seems to be associated with emotional disorders.

**Objectives:**

To discuss an uncommon case of pathological laughter developing after recurrent infarct.

**Methods:**

A 32-year-old male patient presented to the medical emergency for complaints of slurring of speech since 7 hours. On examination, patient was alert, oriented, with blood pressure 150/90 mmHg. He had a history of similar stroke 2 years prior to current complaints and was on treatment for hypertension since then.

Baseline investigations were done. MRI brain revealed *acute lacunar infarcts in bilateral ganglio-capsular region*, chronic small vessel ischaemic changes in B/L periventricular white matter (Fazeka grade 2) and micro-haemorrhages in various brain regions. Patient was managed conservatively (antiplatelets, statins, antihypertensives).

Patient was then referred to Psychiatry department for uncontrollable laughing spells, which started few hours after onset of above complaints. These occurred without any provocation, every 1-2 hours, lasting for several seconds to a minute, and relieved spontaneously. Patient was aware of episodes and found them embarrassing socially. Mental status examination revealed no mood features or other abnormalities.

Patient was prescribed Escitalopram, but shifted to homeopathic medicine and was lost to follow up. Telephonic interview one year later revealed that while other complaints have remitted, patient still has laughing spells of similar quality and frequency.

**Results:**

Discussion: In post stroke PLC, pathological crying represents about 80% cases, while Pure Pathological Laughing, as in the present case, is uncommon. It is generally seen in diffuse CNS pathologies (eg. multiple sclerosis) or bilateral – ischaemic or degenerative.

In case of strokes, PL may herald symptom onset, or may immediately follow focal deficits. The aetiology of PLC is unknown; monoaminergic neurotransmission may be altered in post stroke PLC. SSRIs are regarded as first choice treatment agents, given their greater tolerability overall.

**Conclusions:**

Pathological laughter is a comparatively uncommon but recognisable and treatable post stroke sequela, more commonly seen in bilateral lesions. Patients often describe PL as distressing and socially disabling, but awareness about this condition and available treatments is lacking.

**Disclosure of Interest:**

None Declared

